# The effects of exercise training on Kinesin and GAP-43 expression in skeletal muscle fibers of STZ-induced diabetic rats

**DOI:** 10.1038/s41598-021-89106-6

**Published:** 2021-05-05

**Authors:** Masoud Rahmati, Seyed Jalal Taherabadi

**Affiliations:** Department of Physical Education and Sport Sciences, Faculty of Literature and Human Sciences, Lorestan University, Khoramabad, Iran

**Keywords:** Stem cells, Diseases

## Abstract

Kinesin-1 and Growth Associated Protein 43 (GAP-43) localization in muscle fiber are crucial for proper skeletal muscle hypertrophy. To evaluate this assumption, we investigated the beneficial effects of endurance training on GAP-43 and Kinesin Family Member 5B (KIF5B) expression in *gastrocnemius* muscle of streptozotocin (STZ)-induced diabetic rats. Fifty-two male rats were randomly divided into four groups: healthy control (C), healthy trained (T), diabetic control (DC) and diabetic trained (DT). Diabetes was induced by a single intraperitoneal injection of STZ (45 mg/kg). The rats in DT and T groups were subjected to treadmill running for 5 days a week over 6 weeks. The results indicated that the GAP-43 and KIF5B protein levels in the DC group were significantly lower than those in the C group. Additionally, chronic treadmill running in diabetic rats was accompanied by significant increase of GAP-43 and KIF5B protein expression, compared to DC group. Furthermore, the endurance training in healthy rats was associated with a significant increase of GAP-43 and KIF5B protein levels. In addition, we found positive correlation between GAP-43 and KIF5B protein levels and myonuclear number per fiber and average *gastrocnemius* cross-sectional area (CSA). GAP43 and KIF5B protein levels were decreased in skeletal muscles of diabetic rats, and exercise training had beneficial effects and could restore their abnormal expression. Moreover, there is a strong relationship between muscle hypertrophy and GAP-43 and KIF5B protein levels.

## Introduction

Diabetes mellitus is a group of metabolic diseases associated with numerous systemic complications on several organs. Diabetic myopathy is a pathological change of skeletal muscle in response to the diabetic hormonal/hyperglycemic environment and is characterized by loss of muscle mass and strength^1^. The literature clearly shows that uncontrolled diabetic environment is associated with reductions in skeletal muscle structural, functional, and metabolic capacities^2^; however, the molecular processes governing these changes are still poorly elucidated.

Kinesin-1 (KIF5B) is a microtubule-based motor protein which transports different organelles and macromolecular complex. The KIF5B in muscle cells is related to the anterograde mitochondrial movement^3^ and myofibril assembly^4^. Furthermore, it has also been observed that KIF5B-dependent nuclear positioning is required for proper skeletal muscle function^5^. Loss of KIF5B in skeletal muscles results in defects in localization and positioning of nuclei and causes several muscle dysfunction^6^, suggesting that normal nuclear positioning is critical for skeletal muscle health^7^. Consequently, we speculated that myonuclei localization by KIF5B may be involved in the diabetic myopathy. Growing evidence indicates that KIF5B-driven axonal transport is impaired in diabetes mellitus, probably contributing to the development of subsequently diabetic neurological complications^8^. In our previous study, we observed that diabetic state increases spinal cord KIF5B mRNA expression and sciatic nerves KIF5B content, which was attributed to the hyperglycemic condition^9^. Although, the effect of hyperglycemia on KIF5B expression in skeletal muscle fiber is not elucidated yet, KIF5B might be a possible marker of muscle impairment in diabetes.

Growth Associated Protein 43 (GAP-43), also known as neuromodulin, abundantly expressed in skeletal muscle fibers and regulates calcium handling^10^. It has been reported that *Gap*43-knockout mice showed low survival rates, decreased body weight and declined muscle strength^11^. It was also reported that muscle fibers of myopathic patients overexpress GAP-43^12^; however, to the best of our knowledge, there is no data on GAP-43 in skeletal muscles of STZ-induced diabetic rats. Although, studies revealed a marked reduction in GAP-43 immunoreactivity in the hippocampus of STZ diabetic rats^13^ and in the skin nerve fibers of diabetic patients^14^.

Mounting evidence has demonstrated that endurance training (ET) is associated with multiple health benefits for diabetic patients such as glycemic control and blood lipid improvement^2^. So far, only few studies have investigated the effects of ET on skeletal muscle health in diabetic muscles. Although ET is typically associated with aerobic metabolism, it plays a role in muscle health and prevention of atrophy^15^. It has been reported that exercise training could be an effective, non-pharmacological treatment for diabetes muscle atrophy. Some reports indicate that endurance training by an increase in protein synthesis and inhibition of proteolysis pathway^16^ is an effective therapeutic intervention to prevent muscle atrophy in diabetic patients^17^. Hence, in addition to its beneficial effects on metabolism, ET may be a good intervention to prevent and treat diabetes^3^. However, the exact mechanisms of ET on regulation of skeletal muscle hypertrophy in diabetes remain elusive. We hypothesize that ET has beneficial effects on diabetic myopathy through the maintenance/improvement of muscle mass that might be mediated by GAP-43 and KIF5B. In that context, it was shown that low intensity treadmill exercise promotes GAP-43 levels in injured sciatic nerve of STZ-induced diabetic rats^18^. Similarly, in our previous study, exercise training modulates KIF5B mRNA and KIF5B content to normal levels in spinal cord of diabetic rats^9^. Therefore, we propose that the beneficial effects of ET on diabetes might be related to GAP-43 and KIF5B expression and their co-localization in skeletal muscle.

## Materials and methods

### Animals

A total of 52 adult male Wistar rats were provided from Razi Institute (Karaj, Iran) and housed four-per-cage in an animal lab under standard conditions (12-h light/dark cycle in a room at the temperature of 20–25 °C) with access to food and water ad libitum. All experimental procedures involving animals in this study were reviewed and approved by the Lorestan University Animal Ethics Committee (Reference Number: LUNS.REC.1395.170 at Lorestan University of Medical Sciences) and was according to the NIH guidelines for the care and use of laboratory animals. Additionally, the present study was carried out in compliance with the ARRIVE guidelines. The animals were divided randomly into four groups: (1) healthy control (C, N = 13), (2) control trained (T, N = 13), (3) diabetic control (DC, N = 13) and (4) diabetic trained (DT, N = 13), followed by inducing diabetes in DT and DC groups and performing ET in T and DT groups.

### Diabetes induction

To acclimatize and reach optimal weight (at least 250 g), all the rats were kept in an animal lab for two weeks before the experiments. Subsequently, after an overnight fasting, diabetes was induced through a single intraperitoneal injection of STZ (45 mg/kg; Sigma, St. Louis, MO) solution (dissolved in 0.5 mol/L citrate buffer at pH 4.0). Two days later, diabetes was confirmed by measuring tail vein blood glucose level (> 350 mg/ld.) using an Accu Chek Compact Plus blood glucose meter (Roche Diagnostics K.K., Tokyo, Japan). During the study course, blood glucose levels were controlled once every two weeks.

### Treadmill training protocol

The treadmill training protocol was developed according to previous protocols^19^ and consisted in 6 weeks of moderate-intensity (50–55% of maximal oxygen consumption) endurance aerobic exercise on a leveled motor-driven treadmill (Model T510E, Diagnostic and Research, Taoyuan, Taiwan). The aerobic power of animals in terms of Vo2max was obtained based on the relation of Vo2max to speed and treadmill slope^20^. In the first week, the speed and duration of the treadmill running were 10 m/min and 10 min per day, respectively. The volume of training increased gradually until the fifth week, ending up with training speed and duration of 18 m/min and 30 min per day, respectively. Moreover, as rats were more active in darkness, the front portion of the treadmill lines was covered with a dark thick paper^21^. No electronic shocks were employed to reduce the stress effect of running on treadmill during training sessions. To stabilize the obtained adaptations, training speed and duration were kept constant in the sixth week^20^.

### Measurement of body weight, muscles wet weight and glycosylated hemoglobin (HbA1c)

The rats were weighed (to the nearest 0.1 kg) on a digital balance (model 707; Seca, Hamburg, Germany), at the same time every day for two weeks during conducting the study. After animal scarification, the *gastrocnemius* muscle was removed by a trained researcher and weighed (to the nearest 0.001 g) on a digital balance (model GX-400; A & D Company, Japan). Moreover, whole blood HbA1c level was assessed by enzymatic assay kit (Chrystal Chem, USA).

### Fluorescent immunohistochemistry analysis

For fluorescent immunohistochemistry, *gastrocnemius* muscle tissues were removed and mounted immediately on pieces of cork and fixed with tragacanth gum. Muscles were frozen in isopentane cooled by liquid nitrogen and further stored at  − 80 °C. 10 µm-thick cryosections were labeled against laminin (L9393 Sigma-Aldrich, St. Louis, MO, USA), GAP-43 (ab219582, Abcam, Cambridge, MA), KIF5B (ab167429, Abam, Cambridge, MA) and Pax7 (Developmental Studies Hybridoma Bank, Iowa, IA, USA). Secondary antibodies were coupled to FITC, Cy3 or Cy5 (Jackson Immunoresearch Inc). Finally, slides were stained with DAPI to detect nuclei. To quantify GAP-43, KIF5B and satellite cell abundance, only those cells that were GAP-43^+^, KIF5B^+^ and Pax7^+^ with DAPI^+^ were counted. For confocal analysis, pictures were taken on a TCS SP5 X microscope (Leica Microsystems) at 20X magnification and for each condition of each experiment, at least 10–12 fields chosen randomly were counted. The number of labeled GAP-43^+^, KIF5B^+^ and Pax7^+^ was calculated using the cell tracker in Image J software and expressed as a percentage of total GAP-43^+^, KIF5B^+^ and Pax7^+^. Finally, GAP-43^+^, KIF5B^+^ and Pax7^+^ counts were normalized to fiber number. In order to calculate the percentage of GAP-43-KIF5B colocalization, only those cells that were GAP-43^+^ and KIF5B^+^ with DAPI^+^ were measured and expressed as a percentage of total GAP-43^+^ cells. To measure CSA, images from whole muscle sections were captured at 10X magnification at room temperature using a Carl Zeiss AxioImager fluorescent microscope (Carl Zeiss, Jena, Germany). Muscle fiber CSA was analyzed utilizing Open-CSAM software^22^. Using Image J software, nuclei that clearly resided within the laminin border of the *gastrocnemius* muscle fibers were scored as myonuclei. For this analysis, nine rats in each group had been used and all manual counting was performed by a blinded, well-experienced technician.

### Western blot analysis

Western blot analysis was performed based on our previous study^20^. Polyclonal antibodies for GAP-43 (ab219582), KIF5B (ab167429) and GAPDH (sc-32233) were used. Moreover, data was normalized to GAPDH in the same membrane and expressed as a percentage of control values. For this analysis, four rats in each group had been used.

### Statistical analysis

Statistical analysis was performed using the Graph-Pad Prism statistics software (Graph-Pad Software Inc., San Diego, CA, USA free demo version 5.04). Normality and homogeneity of data were assessed by Shapiro–Wilk and Levene’s test, respectively. A within-between (groups × time) repeated measures ANOVA followed by Tukey’s post hoc test were used to compare differences in blood glucose and body weight between groups through the study period. Two-way analysis of variance (ANOVA) performed to the determining of *gastrocnemius* muscles' weight, GAP-43, KIF5B and Pax7 levels and numbers in experimental groups. Pearson correlation coefficient was computed to assess the correlation between GAP-43, KIF5B and Pax7 protein levels, myonuclear number per fiber, and CSA. Statistical significance level was set at *P* < 0.05. The data was reported as mean ± S.E.M values.

## Results

### Blood glucose levels (BGL), body weight (BW), HbA1c and muscle mass

Diabetes type 1 in animal model showing increased blood glucose levels (BGL) as well as decreased body weight (BW). In this study, after injecting a single dose of STZ into DC and DT animals, the diabetic rats exhibited significant hyperglycemia and decreased BW, and diabetes was successfully induced in all of them (Table 1). As Table 1 shows, although the mean BGL and BW of the rats at the onset of the study were similar, they significantly changed in the animals in DC and DT groups compared to the C group following the first, third and sixth weeks of experiment (*p* < 0.001). In addition, BGL and BW remained consistent throughout the entire of the study in diabetic rats, and compared with DC animals, ET could attenuate it significantly in DT animals (*p* < 0.05). We also measured the HbA1c of rats in different experimental groups after ET period. Compared with the C group, our results showed that diabetes significantly increase HbA1c (Table 1; *p* ≤ 0.001). In addition, performing ET in diabetic rats significantly decreased HbA1c compared with the DC group (*p* ≤ 0.05).

**Table 1:** Blood glucose levels, body weight and muscle mass in different experimental groups.

Groups	Week 0		Week 1th		Week 3th		Week 6th			
	BGL	BW	BGL	BW	BGL	BW	BGL	BW	HbA1c (%)	MM (mg)
C	101.16 ± 1.47	227.97 ± 5.61	100.66 ± 1.63	223.07 ± 4.58	101.16 ± 1.26	232.38 ± 3.52	101.33 ± 1.21	230.49 ± 4.53	5.48	1586 ± 24
T	100.83 ± 1.47	223.07 ± 3.08	101.16 ± 0.0.75	223.81 ± 2.31	100.66 ± 0.51	230.88 ± 3.37	101.83 ± 0.75	226.66 ± 4.90	4.85	1862 ± 51
DC	100 ± 1.54	232.38 ± 3.52	399.83 ± 8.54**	210.69 ± 2.79**	412.66 ± 9.58**	210.55 ± 2.73**	430.5 ± 6.12**	211.87 ± 0.88**	8.64**	1325 ± 45*
DT	99.33 ± 3.14	223.51 ± 4.58	378.66 ± 9.17**	215.08 ± 1.57**	398 ± 10.99**	215.08 ± 1.57**	419 ± 10.91**	216.15 ± 1.27**	6.96*†	1498 ± 37†

C: control; DC: diabetic control; DT: diabetic training; T: control training; BGL: blood glucose levels (mg/dl); BW: body weight (g); MM: muscle mass. Repeated measures ANOVA and two-way ANOVA and Tukey's post hoc test: * and ** showing *p* < 0.05 and *p* < 0.001 versus C, respectively. †*p* < 0.05 versus DC. Thirteen rats in each group were used for this analysis.

We measured the *gastrocnemius* skeletal muscle weight of rats in different experimental groups. Compared with the C group, our results showed that diabetes significantly decrease *gastrocnemius* muscle weight (Table 1; *p* ≤ 0.001). In addition, performing ET in diabetic rats significantly increased *gastrocnemius* muscle weight compared with the DC group (*p* ≤ 0.001). However, there was still a significant difference between the *gastrocnemius* muscle weight of C and DT groups (*p* ≤ 0.039), demonstrating that ET has not been able to fully recover *gastrocnemius* weight loss in diabetic rats (Table 1).

### Muscle size and myonuclear number

**Figure 1:**
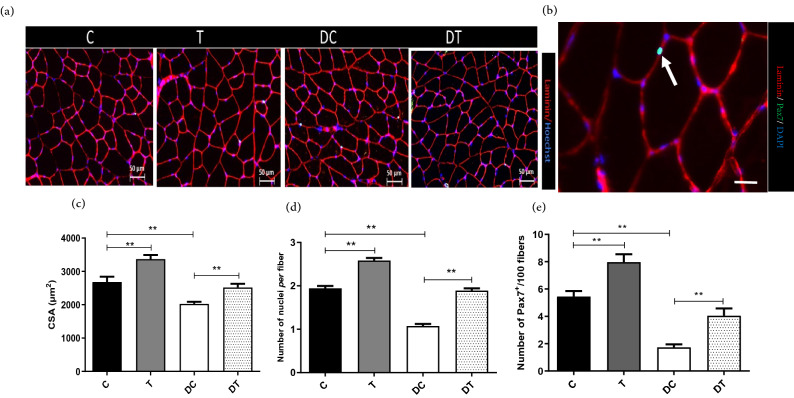
Muscle fiber cross-sectional area (CSA) and satellite cell content in different experimental groups. (**a**) Sections of *gastrocnemius* muscles immunolabeled for Laminin (red) and Hoechst (blue). (**b**) Representative image of satellite cell showing Laminin (red), Pax7 (green) and myonuclei (blue). (**c**) Average *gastrocnemius* muscle CSA and (**d**) myonuclei numbers. (**e**) Number of Pax7^+^/100 fibers. White bar = 50 µm. **p < 0.001. Statistical analysis was performed using the Graph-Pad Prism statistics software (Graph-Pad Software Inc., San Diego, CA, USA free demo version 5.04).

We assessed the magnitude of myonuclear number after ET period. The average muscle CSA in response to diabetes and ET is shown in Fig. 1. The CSA decreased significantly in the *gastrocnemius* skeletal muscle fibers of diabetic rats compared to the C group (*p* < 0.001). In addition, compared to the C group, in the two groups that performed ET (T and DT), CSA was significantly increased (Fig. 1c; *p* < 0.001).

The number of myonuclei in *gastrocnemius* skeletal muscle fibers followed the decrease of CSA in STZ-induced diabetic rats. The muscle nuclei count analysis showed that compared to the C group, the number of *gastrocnemius* muscle nuclei was significantly lower in DC group (Fig. 1d; *p* < 0.01). Moreover, performing ET in T and DT groups were resulted in increase of the number of *gastrocnemius* myonuclei (*p* ≤ 0.001 and *p* ≤ 0.001, respectively).

### Satellite cell content in diabetic myofibers

Further, we decided to quantify the number of satellite cells to determine the cause of lower myonuclear number in *gastrocnemius* muscle of diabetic rats. To test this hypothesis, we further evaluated the number of Pax7^+^ cells in 100 fibers (Fig. 1b). Diabetes in DC group was accompanied with decreased number of Pax7 positive cells significantly in the *gastrocnemius* muscle compared to the C group (Fig. 1e; *p* < 0.001). Moreover, compared to the C group, the number of Pax7^+^ cells were significantly increased in T group (*p* ≤ 0.001 and *p* ≤ 0.001, respectively). Further, compared to the DC group, the number of Pax7^+^ cells were significantly increased in DT group (Fig. 1e; *p* < 0.05) and there was no significant difference between DT and C groups (Fig. 1e; *p* > 0.05).

### GAP-43 and KIF5B protein expression

**Figure 2:**
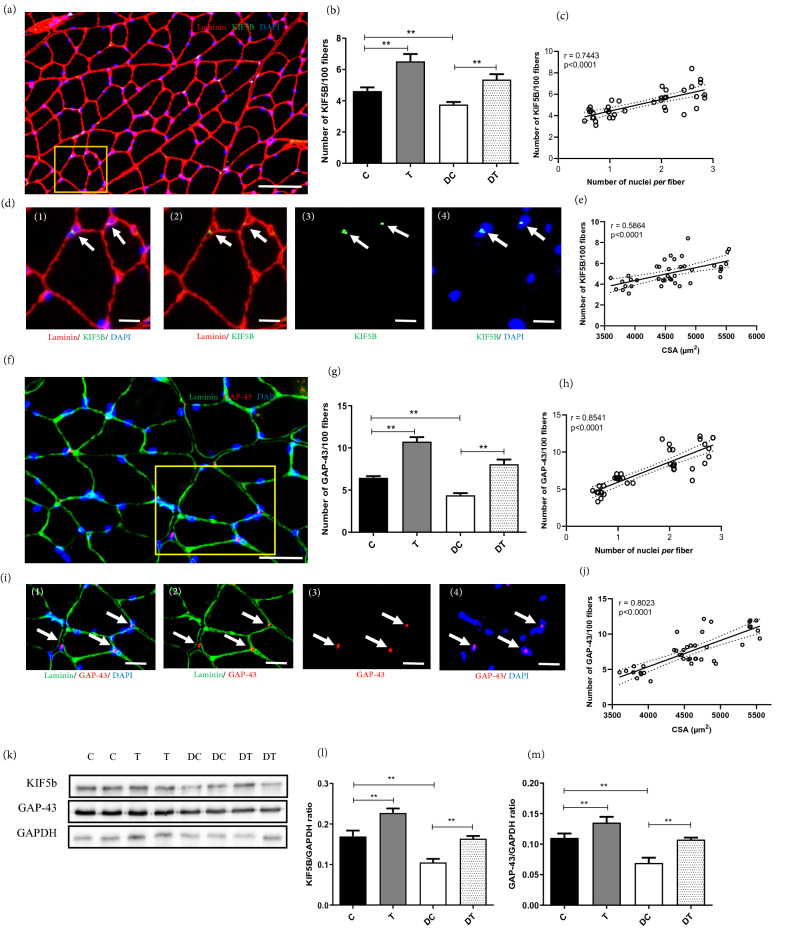
Kif5B and GAP-43 expression in different experimental groups. (**a**) Representative image of Kif5B analysis. (**e**) Number of Kif5B^+^/100 fibers. (**c**) Correlation between Kif5B^+^/100 fibers and the number of nuclei per fiber. (**d** 1–4) Higher magnification of selected area (yellow box) in image (**a**) showing Laminin (red), Kif5B (green) and myonuclei (blue). (**e**) Correlation between Kif5B^+^/100 fibers and average *gastrocnemius* muscle CSA. (**f**) Representative image of GAP-43 analysis. (**g**) Number of GAP-43^+^/100 fibers. (**h**) Correlation between GAP-43^+^/100 fibers and the number of nuclei per fiber. (i 1–4) Higher magnification of selected area (yellow box) in image (f) showing Laminin (green), GAP-43 (red) and myonuclei (blue). (**j**) Correlation between GAP-43^+^/100 fibers and average *gastrocnemius* muscle CSA. (**k**) Representative Western blot images from different experimental groups. Expression of Kif5B (**l**), and GAP-43 (**m**) further demonstrated by Western blotting normalized to GAPDH. White bar = 50 µm. **p < 0.001. Full gel-images are available in Supplementary Material. Statistical analysis was performed using the Graph-Pad Prism statistics software (Graph-Pad Software Inc., San Diego, CA, USA free demo version 5.04).

We measured GAP-43 and KIF5B protein expression by immunohistochemistry and western blot analyses in the *gastrocnemius* skeletal muscle fibers of rats after 6 weeks of the ET period. Our results demonstrated that GAP-43 and KIF5B protein expression between C, DC, DT and T groups was significantly different, demonstrating that GAP-43 and KIF5B protein levels in the rat *gastrocnemius* skeletal muscle fibers were affected by both ET and hyperglycemia (Fig. 2). In this regard, GAP-43 and KIF5B protein levels in the DC group were significantly lower than those in the C group, indicating that diabetes caused a decreased expression of these proteins in the *gastrocnemius* skeletal muscle fibers (*p* ≤ 0.001 and *p* ≤ 0.001, respectively). Furthermore, performing ET in diabetic rats was accompanied by a significant improvement of GAP-43 and KIF5B protein expression as compared with DC and C groups (*p* ≤ 0.001 and *p* ≤ 0.001, respectively). In other words, ET rescued the GAP-43 and KIF5B level reduction in the *gastrocnemius* skeletal muscle fibers of diabetic rats. Moreover, ET in healthy rats was associated with a significant increase of protein levels of GAP-43 and KIF5B in the *gastrocnemius* skeletal muscle fibers (*p* ≤ 0.001 and *p* ≤ 0.001, respectively).

**Figure 3:**
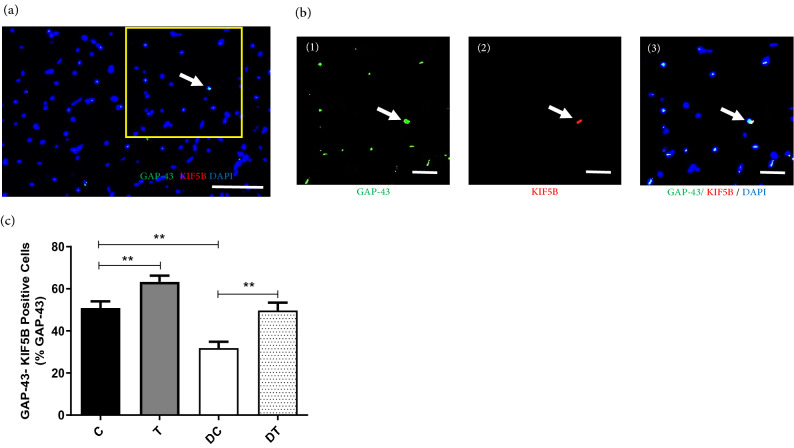
Double-immunostained labeling of KIF5B and GAP43 protein expression in the rat *gastrocnemius* muscle. (**a**) Representative image of KIF5B and GAP43 co-localization analysis showing GAP-43 (green), KIF5B (red) and myonuclei (blue). (**b** 1–3) Higher magnification of selected area (yellow box) in image (**a**). (**c**) GAP-43-KIF5B positive Cells (% GAP-43^+^) in different experimental groups. White bar = 50 µm. **p < 0.001. Statistical analysis was performed using the Graph-Pad Prism statistics software (Graph-Pad Software Inc., San Diego, CA, USA free demo version 5.04).

In addition, there were a strong relationship between KIF5B protein levels and myonuclear number per fiber (Fig. 2c; r = 0.7443, *p* < 0.001); GAP-43 protein levels and myonuclear number per fiber (Fig. 2h; r = 0.8541, *p* < 0.0001); KIF5B protein levels and CSA (Fig. 2e; r = 0.5864, *p* < 0.0001) and GAP-43 protein levels and CSA (Fig. 2j; r = 0.8023, *p* < 0.0001). Finaly, the results of double-immunostained labeling of KIF5B and GAP-43 protein expression in the rat *gastrocnemius* muscle showed that compared with the C group, diabetes caused a decreased of KIF5B^+^ Cells (% GAP-43^+^) in the *gastrocnemius* skeletal muscle fibers (*p* ≤ 0.001). Furthermore, performing ET in diabetic rats was accompanied by a significant improvement of KIF5B^+^ Cells (% GAP-43^+^) as compared with DC and C groups (Fig. 3; *p* ≤ 0.001 and *p* ≤ 0.001, respectively).

## Discussion

In the present study, we observed that diabetic rats experience decreased average muscle CSA in *gastrocnemius* muscle. Diabetes is characterized by muscle atrophy and muscular weakness as a result of increased proteolysis and decreased protein synthesis which is the final consequences of diabetic myopathy^1^. Likewise, Liu and their colleagues (2018) demonstrate that muscle weights and the average CSA of *tibialis anterior* and *gastrocnemius* muscle were reduced significantly in diabetic *db/db* mice^16^. In addition, several studies have shown beneficial effects of treadmill exercise training on decreased fiber CSA in diabetic rodent models^23,24^. In addition, according to Liu et al. study, the average CSA of *tibialis anterior* and *gastrocnemius* muscles were increased significantly in *db/db* mice after moderate treadmill exercise training^16^. Interestingly, it has been reported that the body weight and CSA of the myofibrils and fibers of the diaphragms of the neonatal GAP-43^−/−^ mice were smaller compared to those of the wild-type and GAP-43^+/−^ mice^11^. In agreement with this findings, in the present study we found lower levels of GAP-43 in atrophied *gastrocnemius* muscle in diabetic animals. These data suggest that the level of GAP-43 is a critical factor for skeletal muscle mass and size. However, the exact mechanism of muscle hypertrophy through exercise training and the role of GAP-43 in diabetes remains unclear.

In the present study we observed that diabetes reduces myonuclei content and CSA in *gastrocnemius* muscle of STZ-induced diabetic rats. It has been reported that diabetes causes skeletal muscle atrophy and loss of myonuclei^25^, although underlying mechanisms remain unknown. Likewise, Ato and their colleagues (2019) report that type 2 diabetes mellitus (T2DM) deteriorated fiber CSA and number of myonuclei in rats compared with healthy control rats, which this decrement might be part of a mechanism inducing muscle atrophy in T2DM. They also observed a significant correlation between myonuclear number and fibre CSA in both T2DM and healthy rats^25^. In the present study, next we decided to quantify the number of satellite cells to determine the cause of lower myonuclear number in *gastrocnemius* muscle of STZ-induced diabetic rats and we found lower pax7 positive cells in these animals. In accordance with these findings, muscle stem cells dysfunction had been observed in skeletal muscle of diabetic animals^26^. Satellite cells are essential source of myonuclei and supply additional myonuclei for muscle hypertrophy^27^, while their dysfunction might partly be the cause of muscle atrophy in diabetes^28^. These finding suggest that *gastrocnemius* muscle atrophy in STZ-induced diabetic rats is related to lower nuclear number which might be caused by failed nuclear accretion from satellite cells, while treadmill running exercise thereby restoring satellite cell numbers is capable to produce significant physiological hypertrophic stimuli.

We also observed a possitive relatioship between myonuclear numbers with fiber CSA after ET in *gastrocnemius* muscle. It has been reported that myonuclei are necessary for adaptive hypertrophy and myofiber growth throughout the exercise regimen^29^. It is well documented that the myonuclear accretion is nessecary during skeletal muscle hypertrophy and it might serve other purposes such as muscle repair and remodeling^29^. However, only few studies so far have investigated the effects of exercise training on the myonuclear accretion in diabetic states. For instance, Ato et al. (2019) have investigated the effect of resistance training (RT) on myonuclear content in T2DM rats skeletal muscle and reported that RT significantly increased myonuclear content and show a positive relationship between myonuclear number and fibre CSA^25^. They also obserevd that RT-mediated muscle mass gain and myonuclear accretion were similar in diabetic and healthy rats. According to our results, it can be argued that ET could improve myonuclear accretion in diabetic muscle accompanying with muscle hypertrophy. In the present study, we confirmed that myonuclear number was significantly correlated with fibre CSA in both diabetic and healthy rats. Althought, the underlying mechanisms and physiological significance of ET on the myonuclear accretion in diabetes model remains unknown.

The results of present study point to the fact that diabetes decreases GAP-43 protein levels in *gastrocnemius* muscle. Although GAP-43 evaluation has not been studied in skeletal muscle fibers of diabetic rats, several studies have indicated that this protein undergoes major changes in diabetes state. For example, Maeda et al. (1996) reported that mRNA for GAP-43 levels in dorsal root ganglia was lower in diabetic than in control rats^30^. Zhou et al. (2007) demonstrated that GAP-43 expression decreased in the hippocampal CA1 area and dentate gyrus in diabetic rats^13^. Renno et al. (2012) observed that induction of diabetes for 30 days resulted in decreased expression of GAP-43 in various CNS regions^31^. These findings suggest that GAP-43 plays a crucial role in the physiological functioning of the nervous system, and its abnormal expression is associated with various disorders. However, there is no finding related to GAP-43 changes in skeletal muscles in diabetes. Since it was proposed that GAP-43 was involved in regulation of the calcium handling of skeletal muscles, in our study, the GAP-43 decreased levels might be related to diabetic-induced calcium handling impairments. In line with this hypothesis, previous studies reported deficiency of the skeletal muscle calcium handling in STZ- induced diabetic rats in animals model^32^ and diabetic patients^10^. Nonetheless, in the present study, calcium handling was not evaluated, and evaluation of the relationship between impaired calcium homeostasis and GAP-43 in skeletal muscles needs further investigation.

Our other finding was that diabetes significantly decreased the KIF5B protein levels in *gastrocnemius* muscles. Based on the author’s knowledge, muscular KIF5B expression in diabetes has not been investigated yet, but it has been reported that KIF5B in skeletal muscles is responsible for protein transportation. For instance, Wang et al. (2013) reported that rats with KIF5B conditionally knocked out in myogenic cells show aggregation of actin filaments and intermediate filament proteins in differentiating skeletal muscle cells^4^. In the study conducted by Argyropoulos et al. (2009) silencing KIF5B with siRNA resulted in reduction of endogenous expression of KIF5B in the heart muscle, which is accompanied by a reduction of mitochondrial fluorescence in transfected cells^33^. These scant findings demonstrate that decreased levels of KIF5B in muscles might cause several prominent disruptions. Determining the relationship between KIF5B, mitochondrial dysfunction and aggregation filaments in diabetes is unclear, and it is an interesting topic for future studies.

Remarkably, we found a significant correlation between KIF5B and myonuclei in *gastrocnemius* muscle, which may confirm myonuclear positioning mediated by KIF5B^34^. It was also observed that the KIF5B and myonuclei expression, experience similar reduction and elevation in diabetic states and trained regimen. In line with our assumption, it was reported that defects in myonuclear positioning is crucial in skeletal muscle cells and its mispositioning was associated with muscular dystrophy and cardiomyopathy^25^. Myonuclear positioning is a KIF5B dependent manner, which its impairments has been noted in many muscle diseases^35^. With considering alteration of KIF5B which was correlated with myonuclei number and fiber CSA in the *gastrocnemius* muscle of diabetic and trained rats, it can be hypothesized that myonuclear positioning by KIF5B is impaired in diabetic myopathy and ET could improve these abnormalities, whereas proof of this claim needs further investigation.

The evidence shows that in STZ-induced diabetes, myostatin/protein kinase B (Akt)/ mammalian target of rapamycin (mTOR) and forkhead box protein O1 (FoxO1) signaling pathway downregulates the expression of glucose transporter 4 (GLUT4) and induces skeletal muscle atrophy^36^. Additionally, it has been suggested that Kinesin motor protein directs the translocation of GLUT4 to the plasma membrane in response to insulin^37^. Further, the positive effects of exercise training on GLUT4 expression and insulin resistance improvement is well documented, in both muscle^38^ and adipocytes^39^. Taken together, these studies implicate kinesin motor protein as potential modulator of skeletal muscle atrophy. Further studies are needed to examine the direct effect of kinesin and the role of exercise training in controlling fiber CSA in the skeletal muscle of STZ-induced diabetic rats.

Interestingly, our results of double-immunofluorescence labeling in the present study demonstrate KIF5B-GAP-43 co-localization in skeletal muscle of diabetic rats. However, future studies will reveal the role of this transportation, especially in diabetic conditions. The results of present study provided notably useful insights into the positive relation between GAP-43 expression with myonuclei number and fiber CSA in the *gastrocnemius* muscle of diabetic and trained rats. Based on the previous study^40^, we speculated that GAP-43 may has myogenic effects on skeletal muscle, but the proof of this claim remains as a noteworthy topic for future studies. The effects of ET on GAP-43 and KIF5B in skeletal muscle is sparse. For instance, Tsai et al. (2012) investigated the GAP43 gene expression in rat *gastrocnemius* and observed that treadmill running upregulates myogenesis and muscle strength following botulinum toxin injection. Hence, GAP43 was proposed as a molecular mechanism of treadmill therapy on neuromuscular atrophy^40^. Consistent with our observation, these findings demonstrated that GAP-43 and KIF5B were affected by ET, and based on its physiologic function, it could be concluded that the elevated GAP-43 and KIF5B protein levels in the *gastrocnemius* muscle of diabetic and non-diabetics rats might be related to improvement of muscle hypertrophy, but proof of this claim requires further research.

In conclusion, the present study shows that diabetes decreases GAP-43 and KIF5B protein levels in *gastrocnemius* muscle of STZ-induced diabetic rats and as a non-pharmacologic therapeutic intervention, ET can modify it. Additionally, we observed that GAP-43 and KIF5B expression might be related to impaired muscle CSA and myonuclei content, and ET attenuates their abnormal expression and prevents from skeletal muscle atrophy in STZ-induced diabetic rats.

## Supplementary Information

Supplementary Information

## Abbreviations

KIF5B: Kinesin Family Member 5B
GAP-43: Growth associated protein 43
STZ: Streptozotocin
CSA: Cross-sectional area
ET: Endurance training
BGL: Blood glucose levels
BW: Body weight
T2DM: Type 2 diabetes mellitus
