# Supplementary figures and images for: The effects of exercise training on Kinesin and GAP-43 expression in skeletal muscle fibers of STZ-induced diabetic rats
Source: Sci Rep. 2021 May 5;11:9535. doi: 10.1038/s41598-021-89106-6 (PMC8099856; doi:10.1038/s41598-021-89106-6)

Supplementary Material: Full gel-image of Figure 2k

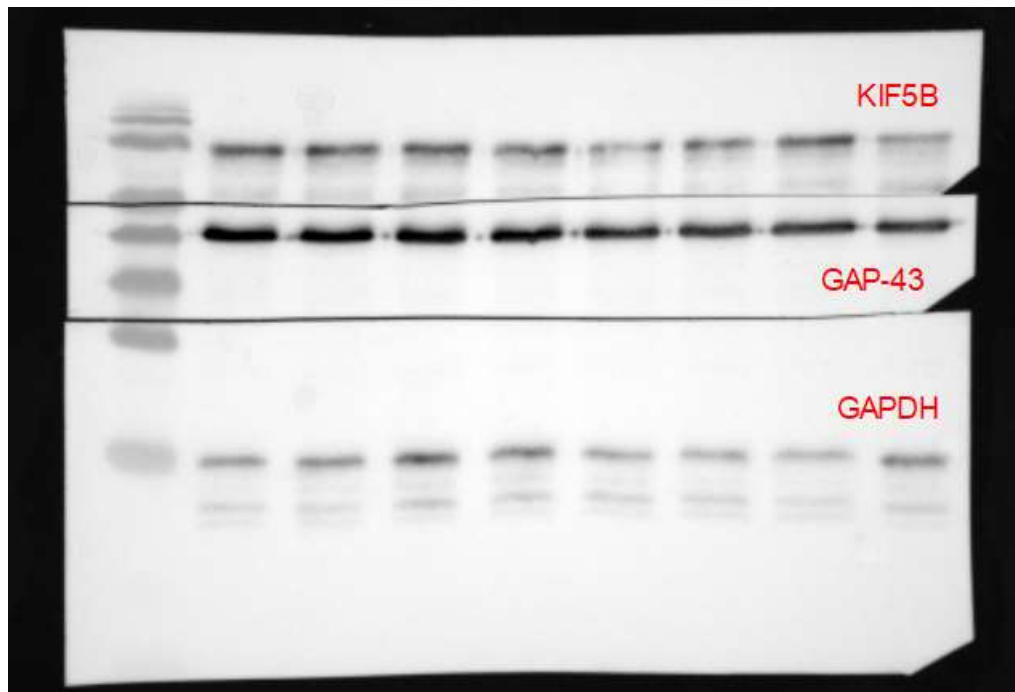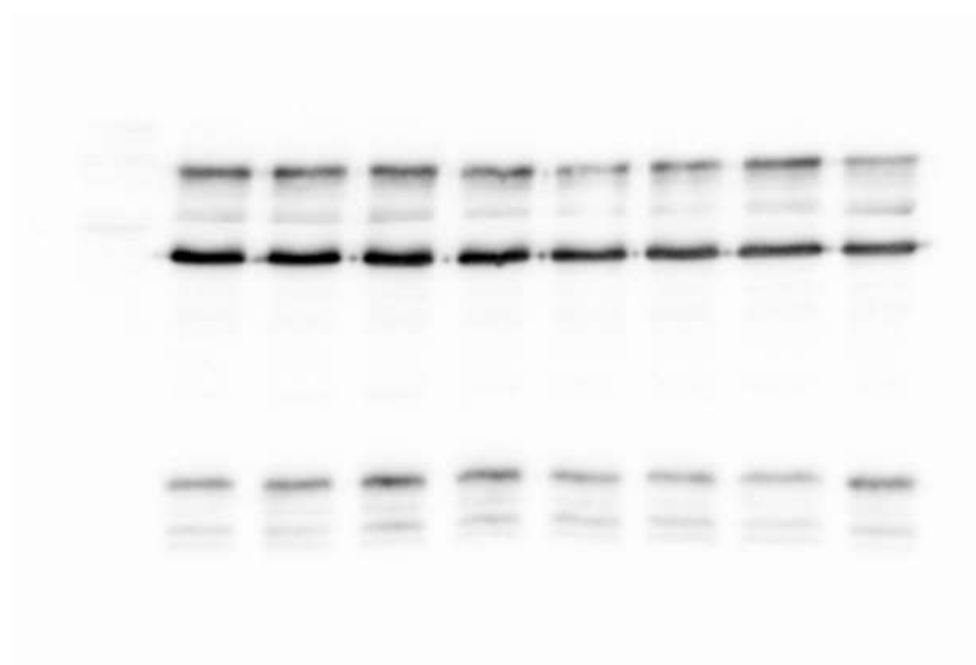

Supplement: Supplementary file 1 — Supplementary Information [file 41598_2021_89106_MOESM1_ESM.pdf]
